# CEMUSA: a graph-based integrative metric for evaluating clusters in spatial transcriptomics

**DOI:** 10.1093/bioinformatics/btag056

**Published:** 2026-02-09

**Authors:** Jiaying Hu, Yihang Du, Suyang Hou, Yueyang Ding, Jinyan Li, Hao Wu, Xiaobo Sun

**Affiliations:** Department of Biomedical Engineering, Southern University of Science and Technology, Shenzhen 518055, China; School of Statistics and Mathematics, Zhongnan University of Economics and Law, Wuhan 430073, China; School of Information Engineering, Zhongnan University of Economics and Law, Wuhan 430073, China; Key Laboratory of Systems Health Science of Zhejiang Province, School of Life Science, Hangzhou Institute for Advanced Study, University of Chinese Academy of Science, Hangzhou 310024, China; Faculty of Computer Science and Control Engineering, Shenzhen University of Advanced Technology, Shenzhen 518055, China; Faculty of Computer Science and Control Engineering, Shenzhen University of Advanced Technology, Shenzhen 518055, China; Institute of Advanced Computing and Digital Engineering, Shenzhen Institute of Advanced Technology, Chinese Academy of Sciences, Shenzhen 518055, China; Department of Human Genetics, Emory University School of Medicine, Atlanta, GA 30322, United States

## Abstract

**Motivation:**

Spatial clustering is a critical analytical task in spatial transcriptomics (ST) that aids in uncovering the spatial molecular mechanisms underlying biological phenotypes. Along with the numerous spatial clustering methods, there comes the imperative need for an effective metric to evaluate their performance. An ideal metric should consider three factors: label agreement, spatial organization, and error severity. However, existing evaluation metrics focus solely on either label agreement or spatial organization, leading to biased and misleading evaluations.

**Results:**

To fill this gap, we propose CEMUSA, a novel graph-based metric that integrates these factors into a unified evaluation framework. Extensive testing on both simulated and real datasets demonstrate CEMUSA’s superiority over conventional metrics in differentiating clustering results with subtle differences in topology and error severity, while maintaining computational efficiency.

**Availability and implementation:**

The source code and data are freely available at https://github.com/YihDu/CEMUSA. CEMUSA is implemented as an R package at https://yihdu.github.io/CEMUSA.

## 1 Introduction

**Figure 1:**
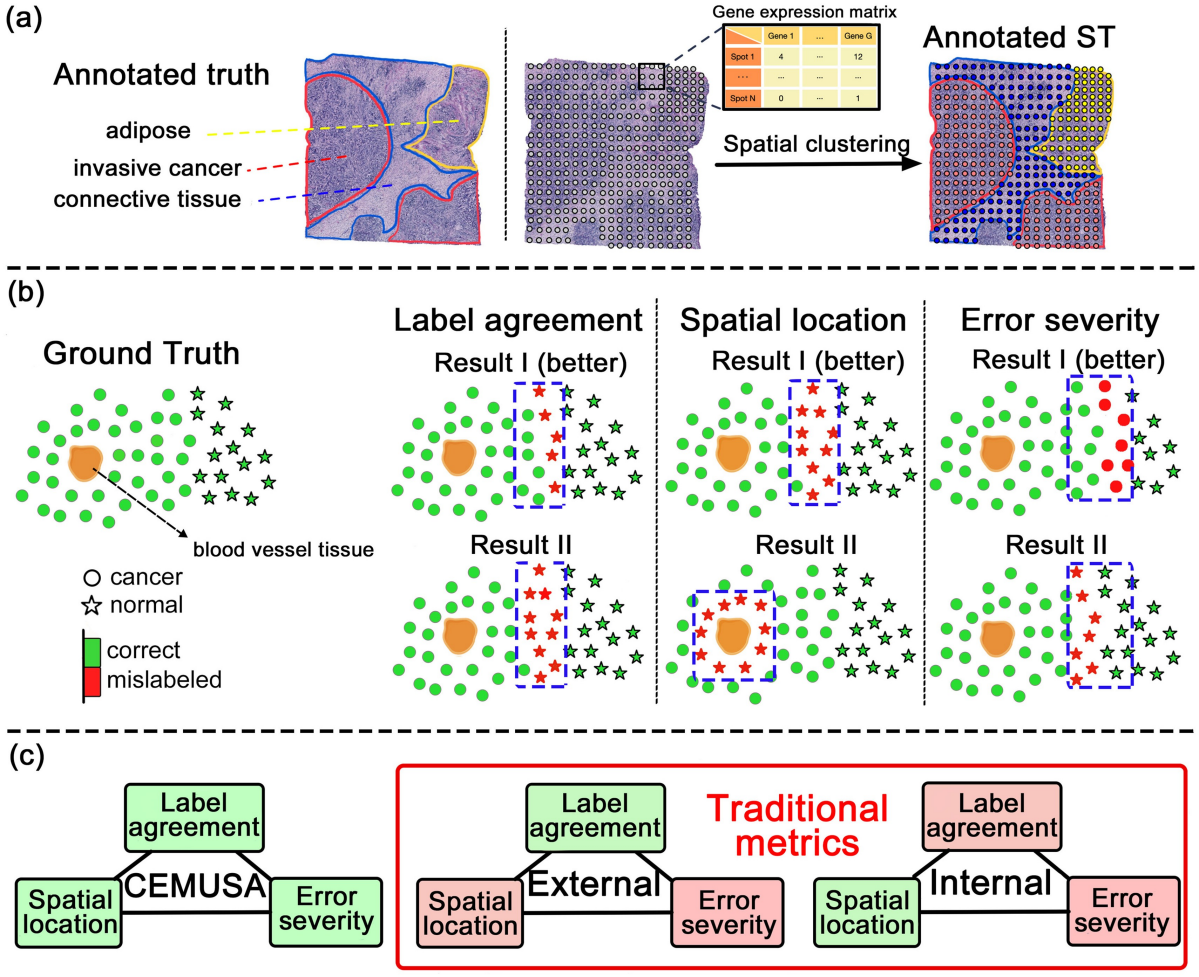
Study overview. (a) The spatial clustering task in spatial transcriptomics (ST) involves measuring gene expression profiles at fixed spots across a tissue slice, based on which the spots are grouped into clusters corresponding to distinct tissue domains. (b) The toy examples illustrate the three factors in clustering evaluation, with Result I superior to Result II. In the label agreement case, result quality improves with fewer misclassified spots. In the spatial location case, misclassifications of malignant tumors near critical locations (e.g. blood vessels) are riskier than those occurring farther away. In the error severity case, misclassifying tumors as normal (false negatives) is more erroneous than the reverse (false positives) in tumor diagnosis. (c) An unbiased and comprehensive evaluation metric should consider three factors: label agreement, spatial location of mislabels, and error severity. Existing internal metrics focus solely on spatial location, while external metrics only account for label agreement. In contrast, CEMUSA simultaneously accounts for all three factors.

Spatial transcriptomics (ST) enables the characterization of spatial gene expression profiles across tissues, providing unprecedented opportunities to understand spatial gene functions and their interactive networks in biological and pathological processes (Dries *et al.* 2021, Chen *et al.* 2022, He *et al.* 2022, Moses and Pachter 2022, Janesick *et al.* 2023). Spatial clustering, a critical ST-based analytical task, aims to delineate tissue space into biologically distinct domains (Zeng *et al.* 2022). This approach allows for the investigation of gene-level mechanisms underlying cell type differentiation, tissue development, and disease origin and progression (Liu *et al.* 2024). Specifically, spatial clustering, based on the spatial coherence of local gene expressions measured at different locations (spots) across the tissue, divides spots into contiguous regions representing different tissue domains, as illustrated in Fig. 1a.

Numerous methods, represented by SpaGCN (Hu *et al.* 2021), STAGATE (Dong and Zhang 2022), and GraphST (Long *et al.* 2023), have been developed for this task. These methods vary in effectiveness due to their different underlying mechanisms, highlighting the need for an authoritative metric that evaluates clustering results unbiasedly and comprehensively. Such a metric provides a valuable tool for selecting spatial clustering methods that produce high-quality clustering results for downstream biomedical studies.

Three key aspects must be considered: **label agreement**, which measures the correspondence between predicted and true labels; **spatial label organization**, which evaluates the consistency of topological structures; and **error severity**, which assesses the impact of mismatched labels on clustering quality. The significance of the three aspects is illustrated in the toy example in Fig. 1b. The leftmost panel shows that the metric should deteriorate as the number of mislabels increases. The middle panel highlights the need to account for spatial locations of mislabels. For instance, misclassified malignant tumors located near blood vessels (Result II) constitutes a more severe error than misclassifying those farther away (Result I) due to a higher metastatic potential. The rightmost panel emphasizes the necessity of considering error severity, as false negatives (FNs) are more critical than false positives (FPs) in tumor diagnosis. There are three additional examples (see Section 1, available as supplementary data at *Bioinformatics* online) to help explain three aspects.

Currently, the evaluation of spatial clustering results relies on conventional clustering metrics, broadly categorized as external and internal (Yuan *et al.* 2024). External metrics, such as the Adjusted Rand Index (ARI) (Rand 1971), Normalized Mutual Information (NMI) (Cover 1999), Jaccard index (Jolliffe and Stephenson 2012), Fowlkes-Mallows Index (FMI) (Halkidi *et al.* 2001), and V-measure (Rosenberg and Hirschberg 2007), assess clustering results by comparing them against ground truth labels, typically in terms of label agreements and disagreements between pairwise instances. However, a significant drawback of these metrics when applied to ST is their treatment of spots as independent instances, neglecting the essential spatial relationships that determine the quality and sensibility of spatial clustering results. Conversely, internal metrics, including Average Silhouette Width (ASW) (Batool and Hennig 2021), the Clustering Hierarchy of Anomalies and Outliers Score (CHAOS) (Alexandrov and Bartels 2013, Guo *et al.* 2021), Percentage of Abnormal Spots (PAS) (Shang and Zhou 2022), Calinski-Harabasz (CH) index (Caliński and Harabasz 1974), and Davies-Bouldin (DB) index (Davies and Bouldin 1979) are designed for instances without ground truth labels but with defined pairwise distance (e.g. using spatial coordinates). These metrics evaluate clustering quality based on same-label cohesion and distinct-label separation, focusing on the spatial continuity of clustering labels. However, they only consider the spatial organization of clustering labels and fail to match them with the ground truth, leading to biased evaluations due to the irregular shapes, varying sizes, and dispersed distribution of tissue domains in an ST slice. Moreover, both external and internal metrics equally treat misclassified instances of the same type, neglecting varying degrees of error severity. These limitations underscore the need for new metrics that integrate all three aspects, providing a more accurate assessment of spatial clustering quality (Fig. 1c).

## 2 Materials and methods

### 2.1 The framework of the proposed CEMUSA

**Figure 2:**
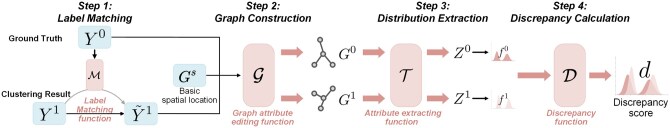
Four steps of CEMUSA. Initially, a matching function is used to match the clustering results to the label space of the ground truth. In the second step, CEMUSA transforms the ground truth and the clustering result into graphs. In the third step, a severity-adjusted edge encoding and a Gaussian kernel density estimation function are used to extract the distribution of the edited graph attributes. Finally, CEMUSA calculates a discrepancy score using a composite function that integrates a sliced Wasserstein distance, a symmetric positive definite exponential kernel function, and a MMD function.

We propose **C**omprehensive **E**valuation **M**etric for **U**nsupervised **S**patial **A**nnotation (**CEMUSA**) to evaluate spatial clustering results from ST data, simultaneously addressing **label agreement**, **spatial label distribution**, and **error severity** (Fig. 1c). CEMUSA’s workflow comprises **four steps** (Fig. 2): Initially, we use a Jaccard coefficient-based function to match the clustering results to the ground truth label space (**Step 1**). Next, CEMUSA converts ground truth and clustering results into undirected graphs (**Step 2**), where nodes represent spatial spots and are positioned according to their locations in the spatial map of the ST dataset. Spatially adjacent nodes with identical labels are connected by edges, which are one-hot encoded based on node label types. Each edge is assigned a weight reflecting the mismatch severity between the connected node pair. In **Step 3**, the severity-adjusted edge encodings from both clustering and ground truth graphs are modeled as manifold distributions in a latent space, with their expected distributional discrepancy serving as a surrogate for labeling inconsistency. Finally, to quantify this discrepancy, CEMUSA uses a composite discrepancy function (**Step 4**) integrating the Sliced Wasserstein distance function, a symmetric positive definite kernel, and the maximum-mean discrepancy (MMD) function. Specifically, edge encoding samples are drawn from the manifold distributions of the clustering result and the ground truth. The sliced Wasserstein distance computes the pairwise discrepancy between samples; while the kernel captures higher-order moments of these discrepancies within a uniquely induced reproducing kernel Hilbert space (RKHS). And the MMD function estimates the expected discrepancy between the clustering result and the ground truth.

### 2.2 Mathematical notations

-
$X\in R^{n\times g}$ represents a ST dataset, where *n* is the number of spots across the tissue section, *g* is the number of genes, and $x_{i,j}$ denotes gene *j’*s expression at location *i*.
-
$Y^{0}\in {\left\{1,2,\ldots ,K\right\}}^{n}$ represents the ground truth labels of the *n* spots, and *K* is the number of domain types.
-
$Y^{1}\in {\left\{a_{1},a_{2},\ldots ,a_{K_{1}}\right\}}^{n}$ represents the cluster labels of the *n* spots, which comprises $K_{1}$ clusters.
-
$G^{0}$ represents the constructed spatial graph of the ground truth.
-
$G^{1}$ represents the constructed spatial graph of the clustering result.
-
$f^{0}$ represents the ground truth graph attribute distribution.
-
$f^{1}$ represents the clustering result graph attribute distribution.

### 2.3 Step 1. Matching the clustering result with the ground truth

We first match the clustering result $Y^{1}$ to the label space of the ground truth $Y^{0}$ using a Jaccard coefficient-based algorithm. Formally, given a cluster with label $u\in \{a_{1},a_{2},\ldots ,a_{K_{1}}\}$ and a ground truth domain with label v∈{1,2,…,K}, we calculate their Jaccard coefficients as:


(1) $$J_{u,v}=\frac{\left|C_{u}\cap C_{v}\right|}{\left|C_{u}\cup C_{v}\right|},J\in R^{K_{1}\times K}$$

where $C_{u}$ denotes the set of spots with label *u*. $J_{u,v}$ represents the similarity between predicted cluster *u* and true domain *v*. When $K=K_{1}$, *J* is input to the Hungarian algorithm (Mills-Tettey *et al.* 2007) to identify the optimal one-to-one correspondence between predicted and true labels. However, when $K\neq K_{1}$, the Hungarian algorithm cannot be applied directly. On one hand, if $K_{1}>K$, *J* is padded with dummy columns of zeros to make it $K_{1}\times K_{1}$. The Hungarian algorithm is then applied to obtain an initial match. Predicted clusters matched to the dummy columns are assigned the true label with which they have the highest Jaccard coefficient. On the other hand, if $K_{1}<K$, *J* is padded with dummy rows of zeros to make it K×K. The Hungarian algorithm is then applied to obtain an initial match. For each true label matched to the dummy rows, we identify the predicted cluster with the highest Jaccard coefficient relative to it. If the spots in the identified cluster are closer to the domain of this true label than to their originally matched domain, they are re-matched to it. After completing this matching process, $Y^{1}\in {\left\{a_{1},a_{2},\ldots ,a_{K_{1}}\right\}}^{n}$ is converted into ${\tilde{Y}}^{1}\in {\left\{1,2,\ldots ,K\right\}}^{n}$, aligning it in the label space of the ground truth. There are two small examples (see Section 2, available as supplementary data at *Bioinformatics* online) to help illustrate our cluster matching process.

Upon completing of this matching, $Y^{1}\in {\left\{a_{1},a_{2},\ldots ,a_{K_{1}}\right\}}^{n}$ is converted into ${\tilde{Y}}^{1}\in {\left\{1,2,\ldots ,K\right\}}^{n}$, which is under the same label space as the ground truth.

### 2.4 Step 2. Transforming the ground truth and clustering result into graphs

After the matching step, we construct undirected and weighted graphs $G^{0}(V,E^{0},W)$ and $G^{1}(V,E^{1},W)$, based on $Y^{0}$ and ${\tilde{Y}}^{1}$, respectively. Here, *V* denotes the set of graph nodes, where each node represents a spot, positioned according to its spatial location. *E* denotes the set of graph edges. *W* denotes the edge weights. The edges are defined as follows:


(2) $$E^{l}(u,v)=\{\begin{array}{l} k,\text{the}\;\text{nodes}\;u\;\text{and}\;v\;\text{are}\;\text{spatially}\;\text{adjacent}\;\text{and}\;\text{with}\;\text{identical}\;\text{label}\;k\\ 0,\text{otherwise} \end{array}$$

where l∈{0,1}.

The edge weight matrix *W* is defined by user and represents an specific assessment of error severity: mistakes having more serious consequences receive larger penalties. For example, *W* can be defined by gene expression similarity and calculated as:


(3) $$W(u,v)=\{\begin{array}{c} \mathrm{Sim}(x_{u},x_{v}),\text{if}\;E(u,v)\neq 0\\ 1-\mathrm{Sim}(x_{u},x_{v}),\text{if}\;E(u,v)=0,\text{and}\;\text{nodes}\;u,v\;\text{are}\;\text{spatially}\;\text{adjacent}\\ 0,\text{otherwise}\; \end{array}$$

Here, $x_{u}$ and $x_{v}$ represent the gene expression profiles of nodes *u* and *v*, respectively. $\mathrm{Sim}(x_{u},x_{v})$ is a function measuring the similarity (e.g. scaled cosine similarity) between the gene expression profiles. The intuition behind this weight definition is to penalize errors more heavily when spots with identical true labels and similar gene expression profiles are assigned to different clusters, and when spots with different true labels and dissimilar gene expression profiles are assigned to the same cluster. We also tested other gene expression measurements (e.g. Euclidean and Poisson/NB-based distances) and found they are effectively equivalent for CEMUSA, yielding nearly identical results. *W* can also be based on an explicit and domain-specific cost. For instance, in clinical diagnostics a false negative is typically more severe than a false positive, because failing to flag a truly malignant region may delay treatment and worsen prognosis. Accordingly, FN should carry a higher weight than FP. We will show an example using this weight in one of the simulations.

This graph construction incorporates information about label agreement, spatial distribution, and error severity, ensuring a comprehensive representation of clustering quality.

### 2.5 Step 3. Extracting distributions of graph attributes

Spatial clustering methods inherently involve randomness, and the observed clustering result $Y^{1}$ merely represents a sample from an unknown underlying distribution $f^{1}$. Therefore, we aim to estimate $f^{1}$ given the constructed spatial graph $G^{1}(V,E^{1},W)$. Formally, we encode graph edge attributes using a one-hot encoding function defined as:


(4) $$z_{i,k}^{1}=\{\begin{array}{c} 1,\text{if}\;\;e_{i}^{1}=k,e_{i}^{1}\in E^{1}\\ 0,\text{otherwise}. \end{array},\forall k\in \{1,2,\ldots ,K\},i\in \{1,2,\ldots ,|E^{1}|\}$$

where $e_{i}^{1}$ represents the *i*th edge in $E^{1}$. After this, we obtain the one-hot encoded edge attribute matrix $Z^{1}∼{\left\{0,1\right\}}^{|E^{1}|\times K}$. For example, given three label types, A, B, and C, the one-hot encoded vector is [1, 0, 0] for type A edge, [0, 1, 0] for type B edge, [0, 0, 1] for type C edge, and [0, 0, 0] for type 0 (i.e. disconnected) edge. Subsequently, $Z^{1}$ is adjusted by the edge weight matrix $\hat{W}={\left[\mathrm{vec}(W)\right]}^{K}\in R^{|E^{1}|\times K}$:


(5) $${\tilde{Z}}^{1}=Z^{1}⊙\hat{W},$$

where ⊙ represents element-wise multiplication. The density $f^{1}$ of edge attribute distribution ${\tilde{Z}}^{1}$ is estimated using a Gaussian kernel density estimator K:


(6) $$f^{1}(x)=\frac{1}{|E^{1}|\times h^{K}}\sum_{r=1}^{\left|E^{1}\right|}K(\frac{x-z_{r}^{1}}{h}),$$

Here, $z_{r}^{1}$ represents the *r*th row of ${\tilde{Z}}^{1}$, *h* is the bandwidth for which a sensitivity analysis is conducted in Section 3.4. Similarly, we estimate the density $f^{0}$ for the ground truth graph following the same process.

### 2.6 Step 4. Computing the discrepancy between graph attribute distributions

To compute the discrepancy between the graph attribute distributions of the ground truth and clustering result, we implement a composite function D, which comprises a sliced Wasserstein distance function W (see definition in Section 3, available as supplementary data at *Bioinformatics* online), a symmetric positive definite exponential kernel function Ξ, and a maximum-mean discrepancy (MMD) function Δ. The sliced Wasserstein distance function measures the discrepancy between two sample distributions of graph attributes. It projects two multivariate distributions to be compared onto multiple random directions, obtaining their 1D representations based on which 1D Wasserstein distance is calculated. The overall distance is computed as the integral of 1D Wasserstein distances over the unit sphere. Since the calculation of 1D Wasserstein distance has closed-form solution, sliced Wasserstein distance greatly improves the computational efficiency for multivariates (Rabin *et al.* 2012, Bonneel *et al.* 2015, Kolouri *et al.* 2016), which is also empirically validated in our complexity analysis in Section 3.4. The kernel function $\Xi {}^{\circ}W^{2}$ is a symmetric positive definite kernel and captures higher-order moments of distributional discrepancy in a uniquely induced reproducing kernel Hilbert space (RKHS), for which we provide a detailed mathematical proof in Sections 3 and 4, available as supplementary data at *Bioinformatics* online. In this uniquely induced RKHS, the MMD function Δ computes the expected discrepancy between the two underlying graph attribute distributions. Put together, we have $D:=(\Delta^{2}{}^{\circ}\Xi {}^{\circ}W^{2})$ and compute the discrepancy between the underlying graph attribute distributions of the ground truth, $f^{0}$, and the clustering result, $f^{1}$, as *d* (Borgwardt *et al.* 2006):


(7) $$\begin{array}{cc} & d\in [0,2]=(\Delta^{2}{}^{\circ}\Xi {}^{\circ}W^{2})[f^{0}||f^{1}]=E_{x,x^{\prime }∼f^{0}}[\Xi (W^{2}(x,x^{\prime }))]\\ & +E_{y,y^{\prime }∼f^{1}}[\Xi (W^{2}(y,y^{\prime }))]-2E_{x∼f^{0},y∼f^{1}}[\Xi (W^{2}(x,y))], \end{array}$$

where $x,y\in R^{K}$. *d* is then reduced to:


(8) $$\begin{array}{cc} & d=\\ & \frac{1}{n_{0}^{2}}\sum_{i=1}^{n_{0}}\sum_{i^{\prime }=i+1}^{n_{0}}\Xi (W^{2}(x_{i},x_{i^{\prime }}))+\frac{1}{n_{1}^{2}}\sum_{j=1}^{n_{1}}\sum_{j^{\prime }=j+1}^{n_{1}}\Xi (W^{2}(y_{j},y_{j^{\prime }}))\\ & -\frac{2}{n_{0}n_{1}}\sum_{i=1}^{n_{0}}\sum_{j=1}^{n_{1}}\Xi (W^{2}(x_{i},y_{j})), \end{array},$$

(9) $$\Xi (W^{2}((x_{i},x_{i^{\prime }}))=\text{exp}\;\left(-\gamma W^{2}(x_{i},x_{i^{\prime }})\right),\gamma >0$$

where $n_{0}$ and $n_{1}$ represent the number of sampled distributions from $f^{0}$ and $f^{1}$, respectively. A larger *d* value indicates greater discrepancy, reflecting poorer clustering quality.

## 3 Results

### 3.1 Model overview

We compare the evaluation performance of CEMUSA with ten widely used benchmark metrics (Hu *et al.* 2024, Yuan *et al.* 2024) across five scenarios, including both simulated and real experimental settings, involving four ST datasets. The benchmark metrics (see Section 5, available as supplementary data at *Bioinformatics* online) include five external metrics (ARI, NMI, Jaccard index, V-measure, and FMI) and five internal metrics (CHAOS, PAS, ASW, CH index, and DB index). The four ST datasets include the BaristaSeq mouse primary visual cortex (mVISp) dataset (slice 1) (Chen *et al.* 2018), the 10× Visium human dorsal lateral prefrontal cortex (hDLPFC) dataset (slice 151507) (Maynard *et al.* 2021), the 10× Visium human breast cancer (hBC) dataset (slice A1 and H1) (Andersson *et al.* 2020), and the MERFISH mouse hypothalamus (mHypo) dataset (tissue section Bregma-0.04) (Moffitt *et al.* 2018). See Section 6, available as supplementary data at *Bioinformatics* online for datasets details.

### 3.2 Simulations

**Figure 3:**
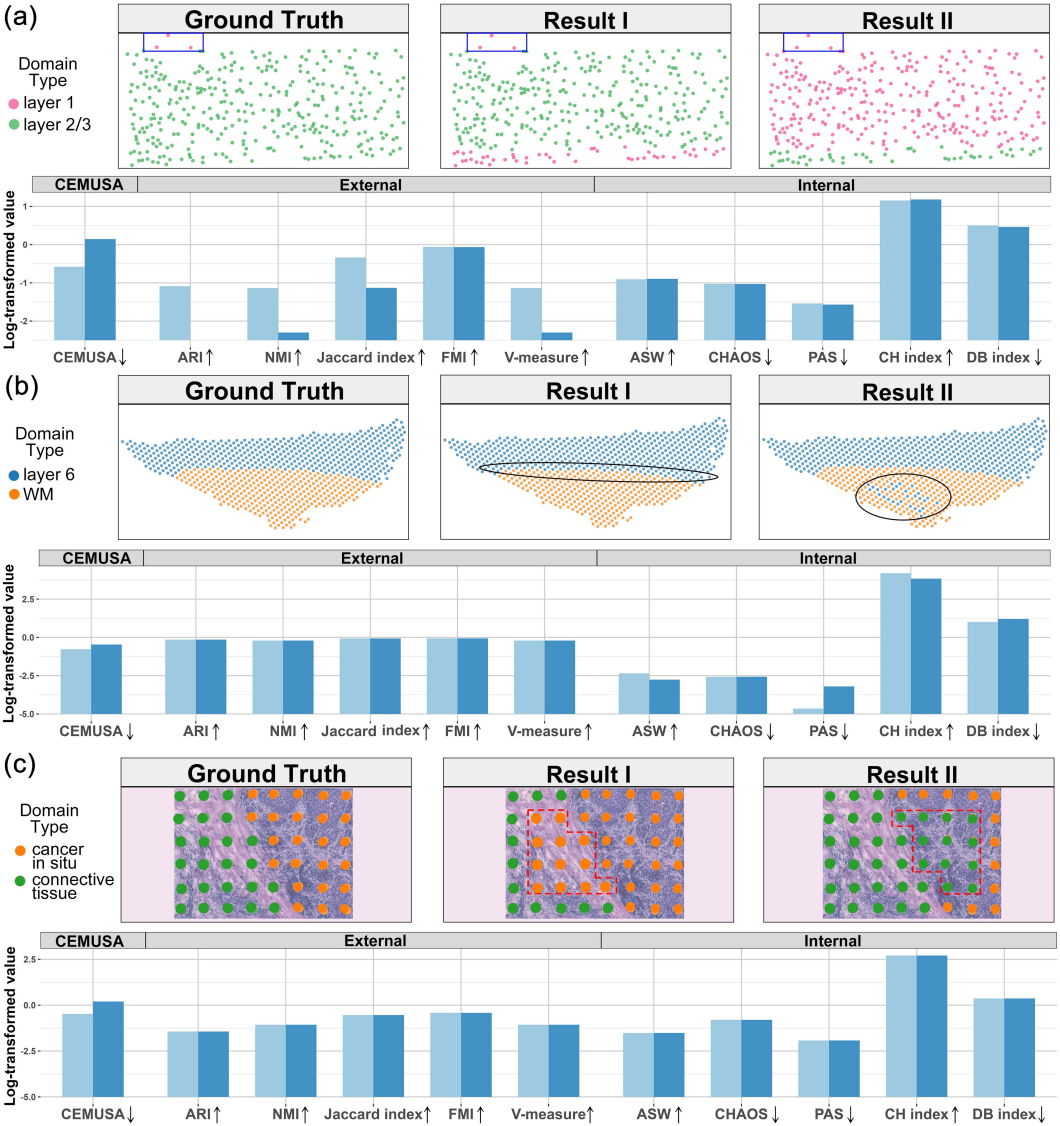
Evaluation of simulated spatial clustering results. (a) Label agreement. Spots in the mouse primary visual cortex layer 1 are shown in red, while those in layer 2/3 indicated in green. The leftmost upper panel represents the ground truth, while the two right panels display simulated results. Result II has more layer 2/3 spots mislabeled as layer 1 than Result I. The log transformed scores of CEMUSA and benchmark metrics are shown in lower panel. The ↑ next to a metric’s name indicates that its value increases with the result quality, and vice versa. (b) Spatial location of mislabels. The leftmost upper panel represents the ground truth labelings of cortex layer 6 (blue) and WM (orange) in the human dorsal lateral prefrontal cortex. In Result I, 29 spots at the boundary between layer 6 and WM are mislabeled as layer 6, whereas in Result II, the same number of spots are mislabeled at the center of WM. The log transformed scores of CEMUSA and benchmark metrics are shown in the lower panel. (c) Error severity of mislabels. Spots in cancer in situ are shown in orange, while those in connective tissue indicated in green. The leftmost upper panel represents the ground truth, while the two right panels display simulated results. Result II has false negative spots while Result I has false positive spots. The log transformed scores of CEMUSA and benchmark metrics are shown in lower panel.

In the first experimental scenario (Fig. 3a), we investigate how evaluation metrics change with varying levels of **cluster label agreement** with respect to the ground truth. For clearer illustration, we crop a partial region from the BaristaSeq (mVISp) dataset (slice 1) derived from the mouse primary visual cortex, which includes three spots from layer 1 (encircled at the upper margin) and 339 spots from layer 2/3. Using this region, we synthesize two results: In result I, 47 spots in layer 2/3 are mislabeled as layer 1, while 292 spots in layer 2/3 are mislabeled as layer 1 in result II (Fig. 3a). The spatial continuity of labels in Result I closely mirrors that of Result II, as they can be viewed with swapped label colors. Consequently, all spatial continuity-oriented internal metrics yield similar scores for both results. However, the presence of the three true layer 1 spots makes Result I superior, since a larger portion of layer 2/3 spots in Result II are mistakenly assigned layer 1 labels. This quality difference is effectively captured by CEMUSA and most external metrics due to their sensitivity to label agreement.

The second experimental scenario (Fig. 3b) focuses on the **spatial location** of mislabels in the 10× Visium human dorsal lateral prefrontal cortex (hDLPFC) dataset (slice 151507). Specifically, in Result I, 29 spots at the white matter (WM) boundary are manually mislabeled as layer 6, while in Result II, an equal number of spots within the core WM region are mislabeled as layer 6. Since peripheral WM shares a greater biological similarity with the adjacent layer 6 than core WM, Result I is superior to Result II. However, all external metrics assign identical scores to both results, as they fail to account for the spatial locations of mislabeled spots. In contrast, CEMUSA and all internal metrics (except CHAOS) correctly distinguish the two results by capturing their topological variations.

The third experimental scenario examines the impact of **mislabeling severity** on result quality using a cropped region from the 10× Visium human breast cancer (hBC) dataset (slice H1) (Fig. 3c). This region encompasses 24 connective tissue spots and 24 cancer *in situ* spots, symmetrically distributed within the region. We synthesize two results from this region, where 12 normal spots are mislabeled as cancer spots (FPs) in Result I, while an equal number of cancer spots are mislabeled as normal (FNs) in Result II. Since the mislabels in both results are symmetric to each other, their topological structures remain invariant. However, in clinical diagnosis, Result I is preferable to Result II, as FNs represent more severe errors than FPs. All internal benchmark metrics fail to differentiate between the two results, as they only consider the topological structure, and so do all external metrics, as they account solely for the number of mislabels, which is identical in both results. In contrast, CEMUSA correctly assigns a superior score to Result I, as it places higher weights to edges connecting cancerous spots than those connecting normal spots.

### 3.3 Real data analyses

**Figure 4:**
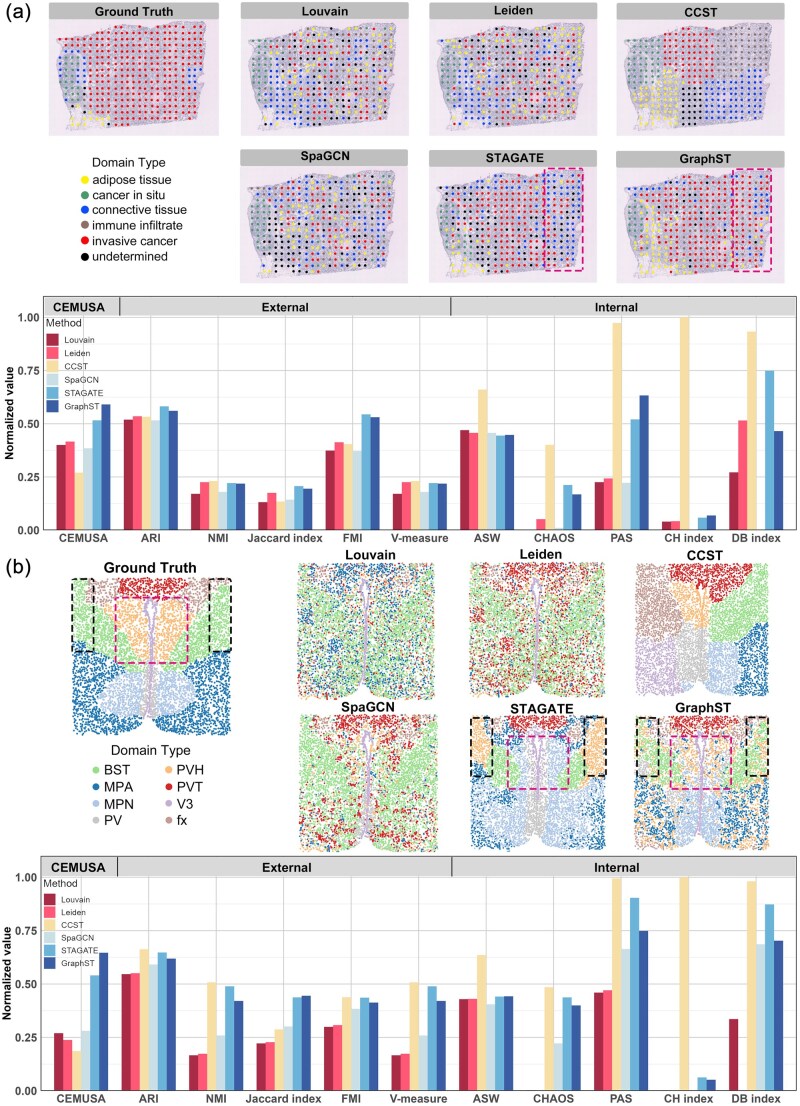
Evaluation of real spatial clustering results. (a) Evaluating clustering results from the 10× Visium human breast cancer (hBC) dataset (slice A1). The upper panels display the ground truth labeling and real results of six spatial clustering methods using the 10× Visium human breast cancer (hBC) dataset (slice A1). The bottom panel presents the normalized scores of CEMUSA and benchmark metrics for these methods. Note that normalized scores always increase with result quality. (b) Evaluating clustering results on the MERFISH mouse hypothalamus (mHypo) dataset. The upper panels display the ground truth labeling and real results of six spatial clustering methods. The red-encircled and black-encircled areas in the STAGATE clustering result highlight regions with significant mislabeling errors. The bottom panel presents the normalized scores of CEMUSA and benchmark metrics for these methods. Note that normalized scores always increase with result quality.

The fourth experimental scenario evaluates real spatial clustering results from the 10× Visium human breast cancer (hBC) dataset (slice A1) generated by six spatial clustering methods (see Section 7, available as supplementary data at *Bioinformatics* online), including Louvain (Que *et al.* 2015), Leiden (Traag *et al.* 2019), CCST (Li *et al.* 2022), SpaGCN (Hu *et al.* 2021), STAGATE (Dong and Zhang 2022), and GraphST (Long *et al.* 2023) (Fig. 4a). Visualizations of these clustering results reveal that STAGATE and GraphST outperform the other four methods in accurately delineating tissue domains. Notably, despite the visually significant misalignment in CCST’s result, all internal metrics rank it first, and all external metrics except Jaccard index rank it in the top three. This outcome is attributed to CCST’s superior spatial continuity of cluster labels—neighboring spots tend to share the same label—driven by CCST’s discriminator, which encourages the embeddings of neighboring spots to be similar to maximize mutual information between local and global graph embeddings. Consequently, internal metrics favor CCST’s results because they prioritize results with spatially continuous labels. External metrics (except Jaccard index), which emphasize pairwise label agreement, fail to adequately penalize CCST’s mislabeling of spots of the same type (e.g. simultaneously mislabeling two spots of type A to type B), as the pairwise agreement among these spots remains unaffected. In contrast, CEMUSA is robust to this limitation and assigns a low score to CCST’s result. Additionally, the visualizations do not clearly distinguish the performance of Louvain, Leiden, and SpaGCN; accordingly, CEMUSA assigns them similar scores, whereas benchmark metrics, such as NMI, CHAOS, DB-index, yield significantly different scores. More importantly, GraphST outperforms STAGATE in terms of **error severity**: STAGATE mislabels more invasive cancer spots as connective tissues in the area encircled by the red rectangle in Fig. 4a, rendering it clinically more erroneous than GraphST. Only CEMUSA and PAS assign higher scores to GraphST compared to STAGATE.

The fifth experimental scenario evaluates real spatial clustering results generated from the MERFISH mouse hypothalamus (mHypo) dataset (tissue section Bregma-0.04) using the same six clustering methods (see Section 7, available as supplementary data at *Bioinformatics* online). The visualizations in Fig. 4b reveal that CCST performs the worst, as its labels are asymmetrically distributed, deviating significantly from the symmetric label distribution in the ground truth. In contrast, GraphST demonstrates the best performance, accurately delineating each tissue domain. However, Fig. 4b shows that most benchmark metrics, particularly internal metrics, rank CCST’s result among the top three. This is because CCST exhibits the best spatial continuity of cluster labels, which internal metrics favor. Additionally, external metrics, which emphasize pairwise label agreement, fail to adequately penalize CCST’s mislabeling of spatial continuous spots of the same type, as pairwise agreement among these spots remains intact. This also explains why benchmark methods assign higher scores to STAGATE compared to GraphST. Specifically, as shown in Fig. 4b, STAGATE mislabels most paraventricular hypothalamic nucleus (PVH) spots as medial preoptic nucleus (MPN) within the red-encircled area and bed nuclei of the stria terminalis (BST) spots as paraventricular hypothalamic nucleus (PVH) in the black-encircled area. In contrast, GraphST’s mislabels are more scattered, so the pairwise agreement is disrupted. Consequently, benchmark metrics, such as ARI and NMI, erroneously assign lower scores to GraphST compared to STAGATE. CEMUSA, however, overcomes these limitations by appropriately accounting for spatial mislabeling and yields rankings of the clustering methods that align closely with visual comparisons of the clustering results against the ground truth.

The sixth and seventh experimental scenarios evaluate real spatial clustering results on the same two datasets (10-hBC-A1 and mHypo) using three recent methods (see Section 7, available as supplementary data at *Bioinformatics* online), including MNMST (Wang *et al.* 2024), SEDR (Xu *et al.* 2024), and Banksy (Singhal *et al.* 2024). Detailed descriptions of these two scenarios are provided in Section 8, available as supplementary data at *Bioinformatics* online.

Collectively, these findings demonstrate that CEMUSA provides the most sensible evaluation, closely aligning with visual comparisons of the clustering results against the ground truth. Detailed results of the four real experimental scenarios are presented in Section 9 Table 3, available as supplementary data at *Bioinformatics* online.

### 3.4 Computational efficiency

#### 3.4.1 Complexity analysis

**Figure 5:**
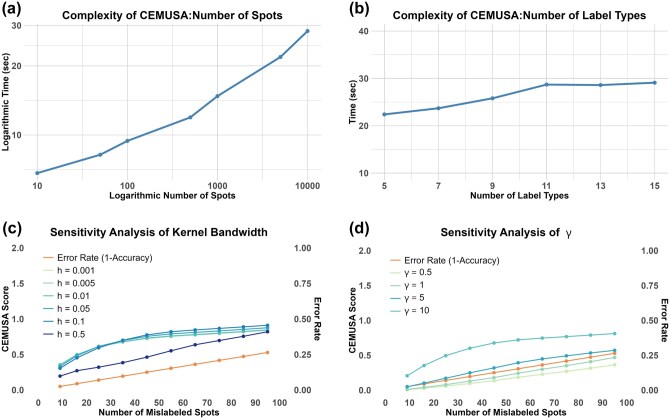
Complexity and sensitivity analysis. (a) Scalability of CEMUSA to input size. The *y*-axis represents computation time in seconds, while the *x*-axis the number of input spots. (b) Scalability of CEMUSA to number of label types. The *y*-axis represents computation time in seconds, while the *x*-axis the number of label types. (c, d) Sensitivity analysis of kernel bandwidth *h* and exponential kernel parameter γ. The left *y*-axis represents CEMUSA scores, the right *y*-axis error rate, and the *x*-axis the number of mislabeled spots. Each line corresponds to a different *h* and γ value, along with a reference error rate line.

To evaluate CEMUSA’s scalability to data size, we conduct a complexity analysis in which the number of spots is gradually increased from 10 to 10 000. Figure 5a shows that CEMUSA is scalable to data size, requiring <100 s to evaluate 10 000 spots. Moreover, we evaluate CEMUSA’s scalability to the number of label types. Using 1000 spots, we start with five label types and incrementally increase the number by two until reaching 15, a relatively large value in ST studies. In each iteration, a spatial clustering result is generated by randomly mislabeling 50% of the spots. As shown in Fig. 5b, CEMUSA maintains good scalability to the number of label types, requiring <30 s for 15 label types.

#### 3.4.2 Sensitivity analysis

We also conduct a sensitivity analysis on the bandwidth parameter *h* in the Kernel Density Estimation [Equation (6)] and the exponential kernel γ parameter [Equation (9)]. Using NetworkX (Hagberg *et al.* 2008), we simulate 360 spatial spots as graph nodes, with gene expression profiles copied from real spots in the 10×-hBC-H dataset. Of these, 180 spots are from the breast gland and the remaining 180 from the cancerous region. We then simulate ten clustering results, progressively increasing the number of mislabeled breast gland spots from 9 to 95. These results are evaluated using CEMUSA with *h* values ranging from 0.001 to 0.5 and γ values ranging from 0.5 to 10, each corresponding to line in Fig. 5c and d. We observe that CEMUSA’s performance remains robust to across different *h* and γ values, consistently aligning with the trend of the reference error rate (1 - accuracy) line. We recommend setting h=0.1 and γ=10 as the default value.

## 4 Discussion

Our study introduces CEMUSA as a novel, integrative metric for evaluating spatial clustering in spatial transcriptomics (ST), addressing key limitations in existing evaluation approaches. The conducted experiments validate our central hypothesis that an effective clustering evaluation metric should incorporate not only label agreement but also spatial organization and error severity to provide a comprehensive assessment of clustering quality.

The results demonstrate that CEMUSA is capable of distinguishing subtle differences in clustering quality that conventional metrics fail to capture. This is particularly evident in cases where existing external metrics, such as ARI and NMI, inaccurately assess clustering performance by treating each spot independently and ignoring spatial continuity. Likewise, internal metrics, which focus on spatial cohesion, fail to account for biologically meaningful label misclassifications. CEMUSA overcomes these limitations by integrating spatial topology and weighting misclassifications based on their severity, making it a more biologically relevant metric for evaluating ST clustering outcomes.

There are some limitations in CEMUSA. The accuracy of CEMUSA greatly depends on how we build the spatial graph that defines spot neighborhoods. If the tissue samples are sparse or irregular, standard graph settings (like the neighborhood radius, the number of neighbors k, tissue-specific connectivity, etc.) can introduce errors and reduce the method’s sensitivity. For these challenging datasets, it is best to use more stable methods for graph construction, such as those based on Delaunay triangulation or adaptive neighborhood sizes, along with routinely checking how the results change with different parameter settings.

In the future, CEMUSA could be expanded in several ways. We could apply it to multi-slice data by first aligning the slices using STAligner (Zhou *et al.* 2023) to construct a 3D structure and then building a graph based on 3D connections. The method can also be improved by factoring in the confidence score for each spot, combining this score with the existing error weight to make the edge weights more accurate. Notably, although it is necessary to first estimate the number of clusters (Chen *et al.* 2023) when ground-truth labels are unavailable, in this setting our metric naturally degenerates to an internal metric. Furthermore, CEMUSA can be adapted to evaluate how accurately cell types are identified in spatial data by combining cell-type hierarchy rules with the spatially aware error-severity weight.

## 5 Conclusion

We introduced CEMUSA, a novel graph-based evaluation metric for assessing spatial clustering quality in ST datasets. By integrating label agreement, spatial organization, and error severity, CEMUSA addresses critical limitations of conventional evaluation metrics and provides a comprehensive framework for evaluating clustering methods. Extensive validation on simulated and real ST datasets demonstrates that CEMUSA effectively distinguishes between clustering results, offering biologically meaningful insights and improving upon existing evaluation techniques.

## Supplementary Material

btag056_Supplementary_Data

## Data Availability

The CEMUSA is implemented as an R package at https://yihdu.github.io/CEMUSA. Several publicly available datasets are used in this manuscript, as summarized in Section 6, available as supplementary data at *Bioinformatics* online.
