# Supplementary material for: CEMUSA: a graph-based integrative metric for evaluating clusters in spatial transcriptomics
Source: Bioinformatics. 2026 Feb 9;42(3):btag056. doi: 10.1093/bioinformatics/btag056 (PMC12960911; doi:10.1093/bioinformatics/btag056)
Supplement: btag056_Supplementary_Data [file btag056_supplementary_data.pdf]

Supplementary Information

Supplementary Section 1. Additional examples to illustrate the three evaluation perspectives

For the label agreement:

We simulate 360 spots (180 type A, 180 type B) and generate ten clusters by progressively flipping A→B from 9 to 95 spots. As the error rate (1–accuracy) increases, CEMUSA also rises, indicating that the metric is highly sensitive to the degree of label agreement and aligns with the decline in labeling correctness relative to the ground truth.

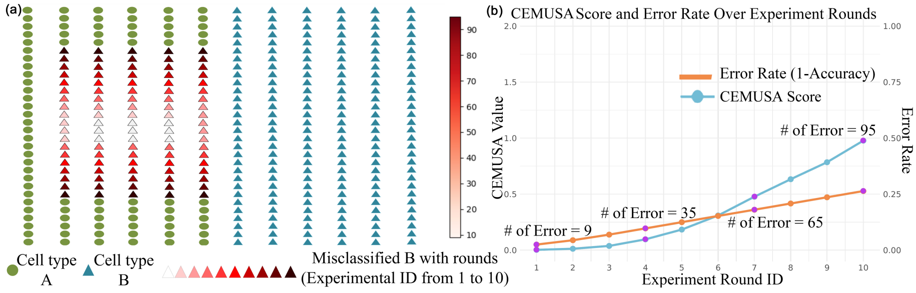

Fig. S1. CEMUSA reflects changes due to increasing mislabels. (a) 180 circle spots and 180 triangle spots represent distributions of type A and type B spots, respectively. The red spots represent type A spots misclassified as type B. The number of misclassified spots range from 9 to 95 in ten sequential experiments. A spot in deeper red is misclassified in later experiments. (b) the trends of SLAM and error rate with increasing mislabelings.

For the spatial label organization:

Aggregated vs. dispersed mislabels carry different spatial semantics even when their counts are identical. To isolate spatial organization, we simulate 100 normal spots as ground truth and construct two clustering results with the same number of errors (40): in Cluster I, the mislabeled cancer spots are dispersed across the section; in Cluster II, the same 40 errors are aggregated into a compact region. Because label agreement is held constant, any quality difference must arise from spatial organization. CEMUSA, unlike purely external accuracy-style metrics, assigns different scores between two clusters, correctly reflecting one topologically implausible error pattern.

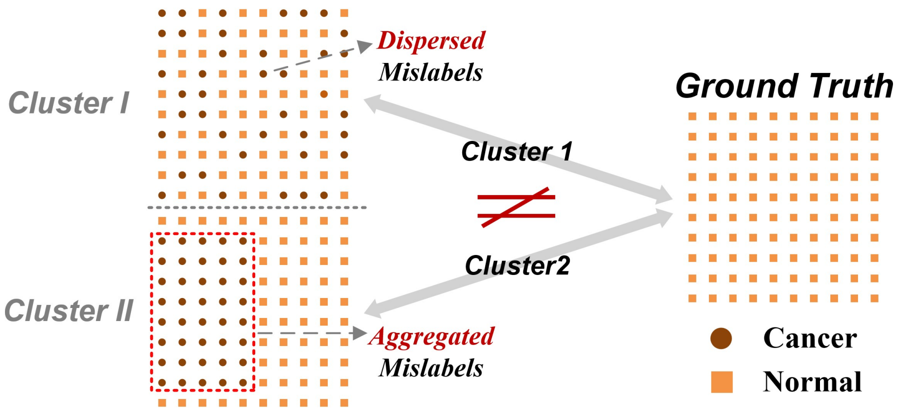

Fig. S2. CEMUSA captures difference in the ground truth between two clusters with aggregated and dispersed mislabels.

For the error severity:

Using the 10x-hBC-H dataset, the ground truth contains 30 spots (10 each from adipose, breast gland, and cancer). We create two clusters with the same error count and mirror-symmetric error locations, so label agreement and spatial topology are held constant. In Cluster I, three cancer spots are mislabeled as breast gland (a far-miss); in Cluster II, three adipose spots are mislabeled as breast gland (a near-miss). Gene expression similarity supports this contrast: average normalized cosine similarity is 0.673 for breast-gland v.s. cancer, but 0.791 for breast-gland v.s. adipose. Thus, the two results differ only in error severity. Because CEMUSA weights edges by inter-class gene similarity, it assigns a better score to Cluster II and a worse score to Cluster I, correctly reflecting that far-miss confusions are more consequential.

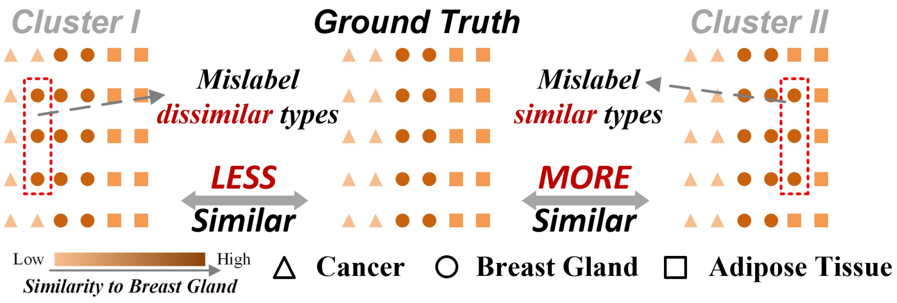

Fig. S3. Triangles, circles, and squares represent breast cancer spots, breast gland spots, and adipose spots, respectively. The color bar indicates the similarity level in gene expressions with the breast gland tissue.

Supplementary Section 2. Matching process example

Here are two small examples to help illustrate our cluster matching process when the number of ground truth labels (K) differs from the number of clusters (K_1).

When $K < K_1$:

We simulate a example which the ground truth has two labels A and B ($K = 2$), while the result has three clusters 1,2, and 3 ($K_1 = 3$).

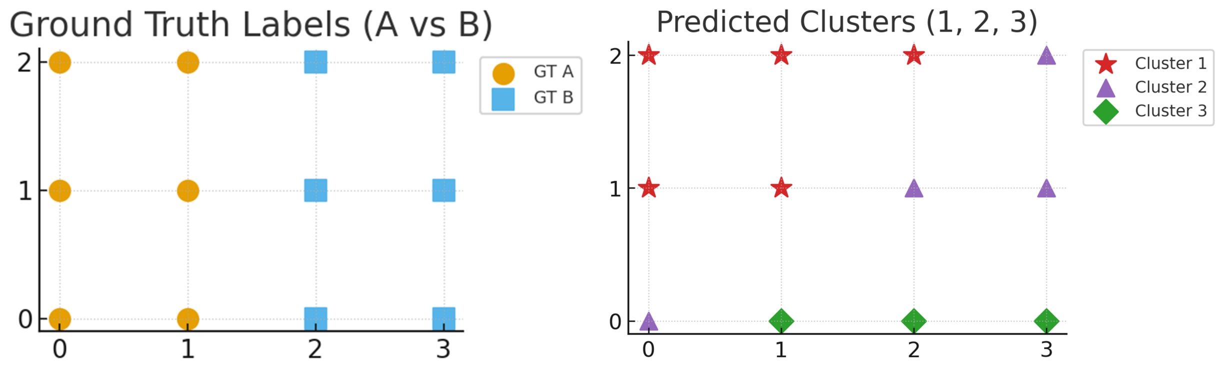

Fig. S4. Illustration of the case $K < K_1$ (more predicted clusters than ground-truth labels): the left panel shows the ground truth with two domains including Label A (orange circles) and Label B (blue squares); the right panel shows the predicted partition with three clusters including Cluster 1 (red stars), Cluster 2 (purple triangles), and Cluster 3 (green diamonds). Axes indicate spot coordinates on the tissue grid.

Table S1. Overlap counts $C_u \cap C_v$ where C_u denotes the set of spots with label u and C_v denotes the set of spots with label v

	**Label A**	**Label B**
**Cluster 1**	4	1
**Cluster 2**	1	3
**Cluster 3**	1	2

Table S2. Jaccard coefficients $J_{u,v}$:

	**Label A**	**Label B**	**Dummy**
**Cluster 1**	$\frac{4}{5+6-4} = 0.57$	$\frac{1}{5+6-1} = 0.10$	0
**Cluster 2**	$\frac{1}{4+6-1} = 0.11$	$\frac{3}{4+6-3} = 0.43$	0
**Cluster 3**	$\frac{1}{3+6-1} = 0.13$	$\frac{2}{3+6-2} = 0.29$	0

Through Hungarian algorithm initial match:

- **Cluster 1** → **Label A** (0.57)
- **Cluster 2** → **Label B** (0.43)
- **Cluster 3** → **Dummy** (0)

Because dummy label does not exist, for Cluster 3:

$$\max\{J_{3,A}, J_{3,B}\} = J_{3,B} = 0.29.$$

So, the final match result is:

•**Cluster 1 → Label A** (0.57)

•**Cluster 2 → Label B** (0.43)

•**Cluster 3 → Label B** (0.29)

When the number of predicted clusters exceeds the number of ground-truth labels, multiple clusters must inevitably be matched to the same true label, yielding a many-to-one mapping.

When $K > K_1$:

We simulate a example which the ground truth has three labels A, B, and C ($K = 3$), while the result has three clusters 1 and 2 ($K_1 = 2$).

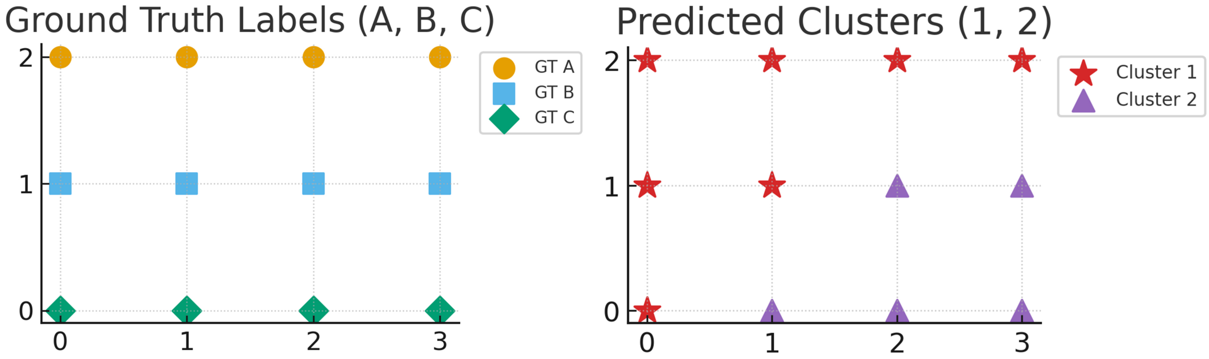

Fig. S5. Illustration of the case $K > K_1$ (fewer predicted clusters than ground-truth labels): the left panel shows the ground truth with three domains including Label A (orange circles), Label B (blue squares), and Label C (green diamonds); the right panel shows the predicted partition with two clusters including Cluster 1 (red stars) and Cluster 2 (purple triangles). Axes indicate spot coordinates on the tissue grid.

Table S3. Overlap counts $C_u \cap C_v$ where C_u denotes the set of spots with label u and C_v denotes the set of spots with label v

	**Label A**	**Label B**	**Label C**
**Cluster 1**	4	2	1
**Cluster 2**	0	2	3

Table S4. Jaccard coefficients $J_{u,v}$:

	**Label A**	**Label B**	**Label C**
**Cluster 1**	$\frac{4}{7+4-4} = 0.57$	$\frac{2}{7+4-2} = 0.22$	$\frac{1}{7+4-1} = 0.10$
**Cluster 2**	$\frac{0}{5+4-0} = 0$	$\frac{2}{5+4-2} = 0.26$	$\frac{3}{5+4-3} = 0.50$
**Dummy**	0	0	0

Through Hungarian algorithm initial match:

•**Cluster 1 → Label A** (0.57)

•**Cluster 2 → Label C** (0.50)

•**Dummy → Label B** (0)

For the uncovered true label B (matched to a dummy row), we select the predicted cluster with the highest Jaccard score to B—Cluster 2, originally aligned with C—and split it by nearest-centroid distance: the two upper spots, being closer to B, are reassigned to B, while the remaining three lower spots remain with C.

Final match result:

•**Cluster 1 → Label A**

•**Three lower spots of Cluster 2 → Label C**

•**Two upper spots of Cluster 2 → Label B**

When the number of ground-truth labels exceeds the number of predicted clusters, the clustering has failed to include all cell types; thus the predicted cluster with the highest Jaccard score should be split so that its constituents can be reassigned to the corresponding true labels.

Supplementary Section 3. Definitions and Properties of Sliced Wasserstein Distance

Definition 1 (**Sliced Wasserstein Distance**). Assume $\mathcal{P}$ and $\mathcal{Q}$ are two continuous d-dimensional probability measures with probability density functions p and q, respectively. Let v denote a random projection direction. The sliced Wasserstein distance between p and q is defined as:

$$\mathcal{W}^2(\mathcal{P}, \mathcal{Q}) := \int_{S^{d-1}} W^2(R(p(.,v)), R(q(.,v)))dv \tag{1}$$

where R is the d-dimensional Radon transform function (Kolouri et al. [2016]) used to project probability densities of $\mathcal{P}$ and $\mathcal{Q}$ onto direction v. W represents the $L2$ Wasserstein distance kernel.

Definition 2 (**Conditionally Negative Definite Kernel**) A conditionally negative definite kernel is a symmetric function k: $\mathbb{S} \times \mathbb{S} \to \mathbb{R}$ that satisfies:

$$\sum_i^N \sum_j^N a_i a_j k(s_i, s_j) \leq 0, \forall s_i \in \mathbb{S}, \forall a_i \in \mathbb{R} \text{ s.t. } \sum_i^N a_i = 0. \tag{2}$$

Properties 1 (Conditionally Negative Definiteness). Sliced Wasserstein distance kernel is conditionally negative definite.

Proof According to Brenier's theorem (Brenier [1991], Kolouri et al. [2016]), $L2$ Wasserstein distance can be computed as:

$$W^2(P, Q) = \int (\mathcal{T}_{\mathcal{P}\to\mathcal{Q}}(x) - x)^2 q(x)dx, \tag{3}$$

where $\mathcal{T}_{\mathcal{P}\to\mathcal{Q}}(x)$ is an unique optimal transport function that maps measure space $\mathcal{P}$ to $\mathcal{Q}$. It is monotonically increasing and satisfies:

$$\mathcal{T}_{\mathcal{P}\to\mathcal{Q}}(x) := \inf_{t\in\mathbb{R}}\{\int_{-\infty}^{t} p(t)dt \geq \int_{-\infty}^{x} q(x)dx\} \tag{4}$$

We define an inner product space $\mathcal{I}$ over functions f, g: $\mathbb{R} \to \mathbb{R}$ as:

$$\langle f, g\rangle_{\mathcal{I}} := \int f(x)g(x)dx \tag{5}$$

and a function:

$$h_{\mathcal{P}\to\mathcal{Q}}(x) := (\mathcal{T}_{\mathcal{P}\to\mathcal{Q}}(x) - x)\sqrt{q(x)} \tag{6}$$

Then we have:

$$\begin{aligned}
&W^2(\mathcal{P}, \mathcal{Q}) = W^2(\mathcal{Q}, \mathcal{P}) = \langle h_{\mathcal{P}\to\mathcal{Q}}, h_{\mathcal{P}\to\mathcal{Q}}\rangle_{\mathcal{I}},\\
&W^2(\mathcal{Q}, \mathcal{Q}) = 0,\\
&W^2(\mathcal{P}, \Pi) = W^2(\Pi, \mathcal{P}) = \int (\mathcal{T}_{\Pi\to\mathcal{P}}(x) - x)^2 p(x)dx\\
&= \int (\mathcal{T}_{\Pi\to\mathcal{P}}(\mathcal{T}_{\mathcal{P}\to\mathcal{Q}}(u)) - \mathcal{T}_{\mathcal{P}\to\mathcal{Q}}(u))^2 \underbrace{p(\mathcal{T}_{\mathcal{P}\to\mathcal{Q}}(u))\mathcal{T}'_{\mathcal{P}\to\mathcal{Q}}(u)}_{p(\mathcal{T}_{\mathcal{P}\to\mathcal{Q}}(u))d\mathcal{T}_{\mathcal{P}\to\mathcal{Q}}(u)=q(u)du} du\\
&= \int (\mathcal{T}_{\Pi\to\mathcal{P}}(\mathcal{T}_{\mathcal{P}\to\mathcal{Q}}(u)) - \mathcal{T}_{\mathcal{P}\to\mathcal{Q}}(u))^2 q(u)du\\
&= \int ((\mathcal{T}_{\Pi\to\mathcal{Q}}(u) - u) - (\mathcal{T}_{\mathcal{P}\to\mathcal{Q}}(u) - u))^2 du\\
&= \langle h_{\Pi\to\mathcal{Q}} - h_{\mathcal{P}\to\mathcal{Q}}, h_{\Pi\to\mathcal{Q}} - h_{\mathcal{P}\to\mathcal{Q}}\rangle_{\mathcal{I}}
\end{aligned} \tag{7}$$

Then we have:

$$\begin{aligned}
&\sum_i^N \sum_j^N a_i a_j W^2(\mathcal{P}_i, \mathcal{P}_j)\\
&= \sum_i^N \sum_j^N a_i a_j \langle h_{\mathcal{P}_i\to\mathcal{Q}} - h_{\mathcal{P}_j\to\mathcal{Q}}, h_{\mathcal{P}_i\to\mathcal{Q}} - h_{\mathcal{P}_j\to\mathcal{Q}}\rangle_{\mathcal{I}}\\
&= \underbrace{2\sum_i^N a_i^2 \langle h_{\mathcal{P}_i\to\mathcal{Q}}, h_{\mathcal{P}_i\to\mathcal{Q}}\rangle_{\mathcal{I}}}_{=0} - 2\sum_i^N \sum_j^N a_i a_j \langle h_{\mathcal{P}_i\to\mathcal{Q}}, h_{\mathcal{P}_j\to\mathcal{Q}}\rangle_{\mathcal{I}}\\
&= -2\langle \sum_i^N a_i h_{\mathcal{P}_i\to\mathcal{Q}}, \sum_i^N a_i h_{\mathcal{P}_i\to\mathcal{Q}}\rangle_{\mathcal{I}} \leq 0
\end{aligned} \tag{8}$$

Thus, for sliced Wasserstein distance, we have:

$$\begin{aligned}
&\sum_{i}^{N}\sum_{j}^{N} a_i a_j \mathcal{W}^2(\mathcal{P}_i, \mathcal{P}_j) \\
&= \sum_{i}^{N}\sum_{j}^{N} a_i a_j \int_{S^{d-1}} W^2(R(p_i(.,v)), R(p_j(.,v)))dv \\
&= -2 \int_{S^{d-1}} \langle \sum_{i}^{N} a_i h_{R(p_i(.,v))\to R(p_j(.,v))}, \sum_{i}^{N} a_i h_{R(p_i(.,v))\to R(p_j(.,v))} \rangle_{\mathcal{I}} dv \\
&\leq 0
\end{aligned} \tag{9}$$

This completes the proof. □

Supplementary Section 4. Definition and Properties of Sliced Wasserstein Gaussian Kernel

Definition 3 (**Sliced Wasserstein Gaussian Kernel**). Sliced Wasserstein Gaussian Kernel is a symmetric function κ: $\mathbb{S} \times \mathbb{S} \to \mathbb{R}$:

$$\kappa(s_i, s_j) := \exp(-\psi \mathcal{W}^2(s_i, s_j)), \forall s_i \in \mathbb{S}, \psi > 0 \tag{10}$$

Properties 2 (**Positive Definiteness and Inducibility of Unique RKHS**). Sliced Wasserstein Gaussian kernel is positive definite and induces a unique reproducible kernel Hilbert space (RKHS).

Proof As proved by C.Berg etc (Berg et al. [1984a,b]):

"A kernel $k(s_i, s_j) = \exp(-\psi f(s_i, s_j)), \psi > 0$ is positive definite $\iff$ f is conditionally negative definite."

Since $\mathcal{W}^2$ is conditionally negative definite (Properties 1), $\kappa(s_i, s_j) = \exp(-\psi \mathcal{W}^2(s_i, s_j))$ is positive definite. Due to Moore-Aronszajn theorem (Aronszajn [1950]), κ, as a symmetric and positive definite kernel, induces a unique RKHS. □

Proposition 1 *Sliced Wasserstein Gaussian kernel captures all moments of the squared sliced Wasserstein distance.*

Proof Using Taylor expansion, we have:

$$\begin{aligned}
\kappa(s_i, s_j) &= \exp(-\psi \mathcal{W}^2(s_i, s_j)) \\
&= \sum_{i}^{+\infty} \frac{1}{i!} (-\psi)^i (\mathcal{W}^2(s_i, s_j))^i,
\end{aligned} \tag{11}$$

which is a linear combination of all moments of squared sliced Wasserstein distance. □

Supplementary Section 5. Benchmark evaluation metrics

External Metrics

- ARI (Adjusted Rand Index): ARI (Rand [1971]) is a metric used to measure the similarity between two clustering results, adjusted for the chance grouping of elements. It compares the number of pairs of elements consistently assigned to the same or different clusters in both partitions, while correcting for expected agreements under random labeling. The ARI ranges from -1 to 1, where 1 indicates perfect agreement, 0 indicates random clustering, and negative values suggest worse-than-random clustering. Let n represents the total number of spots, n_{ij} the number of spots of type i within cluster j, a_i the total number of spots of type i, b_j the total number of spots within cluster j. Then ARI is calculated as follows:

$$\text{ARI} = \frac{\sum_{i,j} \binom{n_{ij}}{2} \left(\sum_i \binom{a_i}{2} \sum_j \binom{b_j}{2}\right) / \binom{n}{2}}{\frac{1}{2}\left[\sum_i \binom{a_i}{2} + \sum_j \binom{b_j}{2}\right] - \left(\sum_i \binom{a_i}{2} \sum_j \binom{b_j}{2}\right) / \binom{n}{2}}. \tag{12}$$

- NMI (Normalized Mutual Information): NMI (Cover [1999]) is a metric used to evaluate the similarity between two clustering results by measuring the amount of shared information. It is based on mutual information and adjusts for differences in cluster sizes, making it robust for comparing partitions with varying numbers of clusters. NMI is normalized to range between 0 and 1,

where 1 indicates perfect agreement between clusters and 0 indicates no shared information. The computation of NMI is defined as:

$$\text{NMI} = \frac{\text{MI(U,V)}}{\sqrt{H(U) \times H(V)}}, \tag{13}$$

where $H(U)$ and $H(V)$ denote the entropy of clusters U and V, respectively, and $\text{MI}(U, V)$ represents the mutual information between them. The mutual information is calculated as:

$$\text{MI}(U, V) = \sum_{i=1}^{|U|} \sum_{j=1}^{|V|} \frac{|U_i \cap V_j|}{N} \log\left(\frac{N\,|U_i \cap V_j|}{|U_i|\,|V_j|}\right), \tag{14}$$

where N is the total number of elements, $|U_i|$ and $|V_j|$ represent the sizes of cluster U_i and V_j, and $|U_i \cap V_j|$ is the number of elements shared between clusters U_i and V_j.

- Jaccard index: The Jaccard index (Jolliffe and Stephenson [2012]) measures the similarity between true labels (C) and clustering results (K) by calculating the ratio of the size of their intersection $|C \cap K|$ to the size of their union$|C \cup K|$ for each category. The final score is obtained by averaging this ratio across all categories. The Jaccard index ranges from 0 to 1, where 1 indicates perfect overlap between the true labels and clustering results, and 0 indicates no overlap. The Jaccard index is computed as:

$$\text{Jaccard index} = \frac{1}{k} \sum_{l=1}^{k} \frac{|C_l \cap K_l|}{|C_l \cup K_l|}, \tag{15}$$

where k denotes the total number of domain types, C_l and K_l represents the true and clustering labels of domain l, respectively.

- FMI (Fowlkes-Mallows Index): The FMI (Halkidi et al. [2001]) evaluates the similarity between two clustering results by assessing the overlap of their pairwise assignments. It calculates the geometric mean of precision and recall based on true positive, false positive, and false negative pairs. The FMI ranges from 0 to 1, where 1 indicates perfect agreement between the two clusterings, and 0 represents no agreement. The FMI is computed as:

$$\text{FMI} = \frac{\text{TP}}{\sqrt{(\text{TP} + \text{FP})\,(\text{TP} + \text{FN})}}, \tag{16}$$

where TP (True Positive) is the number of pairs of points that belong to the same cluster in both truth labels and predicted labels, FP (False Positive) is the number of pairs of points that belong to the same cluster in predicted labels but not in truth labels, and FN (False Negative) is the number of pairs of points that belong to the same cluster in truth labels but not in predicted labels.

- V-measure: The V-measure(Rosenberg and Hirschberg [2007]) is an external clustering evaluation metric that assesses the agreement between predicted clustering and ground truth by balancing two aspects: homogeneity and completeness. Homogeneity ensures that each cluster contains only data points from a single true class, while completeness ensures all data points of a true class are assigned to the same cluster. The V-measure is the harmonic mean of these two components, providing a score between 0 and 1, where 1 indicates perfect clustering. It is calculated as:

$$\text{V-measure} = \frac{2 \times h \times c}{h + c}, \tag{17}$$

where $h = 1 - \frac{H(C|K)}{H(C)}$ is the homogeneity, $c = 1 - \frac{H(K|C)}{H(K)}$ is the completeness, $H(C \mid K)$ and $H(K \mid C)$ are the conditional entropies of the true labels C and the predicted clusters K.

Internal Metrics

- Adjusted Silhouette Width (ASW): The ASW (Batool and Hennig [2021]) is a metric used to evaluate the quality of clustering by measuring how well a spot is assigned to its cluster compared to other clusters, while adjusting for chance. It is based on the Silhouette score, which considers the cohesion (distance within the same cluster) and separation (distance to the nearest other cluster). The adjustment in ASW accounts for differences in cluster sizes and random assignments. ASW values range from -1 to 1, where values close to 1 indicate well-separated and cohesive clusters, and values near 0 suggest random assignments. We first calculate Silhouette Width for spot i as follows:

$$\text{SW}_i = \frac{b_i - a_i}{\max(a_i, b_i)}, \tag{18}$$

where a_i is the average distance to all other spots in the same domain, and b_i is the average distance to all spots in the nearest other domain.

- Clustering Hierarchy of Anomalies and Outliers Score (CHAOS): The CHAOS metric can be applied to assess the spatial clustering performance by measuring the spatial continuity of identified domains (Alexandrov and Bartels [2013], Guo et al. [2021]). We

first build a 1-nearest neighbor (NN) graph, wherein each spot is connected to its closest spot. Then we have:

$$w_{kij} = \begin{cases} d_{ij}, & \text{if spot } i \text{ and spot } j \text{ are connected in the 1NN graph in cluster } k, \\ 0, & \text{otherwise.} \end{cases} \tag{19}$$

where d_{ij} is the Euclidean distance between spots i and j. The CHAOS is then computed as the average value of w:

$$\text{CHAOS} = \frac{\sum_{k=1}^{K} \sum_{i,j}^{n_k} w_{kij}}{N}, \tag{20}$$

where n_k is the number of cells in the k-th domain, N is the total number of spots, and K is the number of domains. CHAOS values range from 0 to N/A, with lower scores signifying greater spatial continuity and thus superior overall performance.

- Percentage of Abnormal Spots (PAS): The PAS metric (Shang and Zhou [2022]) evaluates the spatial uniformity of cluster labels within a ST field. It is defined as the fraction of spots whose cluster labels differ from at least six of their ten nearest neighbors. The PAS scores range from 0 to 1, with a smaller value indicating greater spatial homogeneity in cluster labels.
- Calinski-Harabasz (CH) index: The CH index (Caliński and Harabasz [1974]) is a metric for evaluating clustering quality by measuring the ratio of between-cluster dispersion to within-cluster dispersion. It assesses how well-separated clusters are while ensuring that spots within each cluster are compact. CH index is always positive, with a higher value indicating better-defined and more distinct clusters. Given a dataset of N spots and K clusters, CH index is calculated as:

$$\text{CH} = \frac{\frac{1}{K-1} \sum_{k=1}^{K} n_k \left\| c_k - c \right\|^2}{\frac{1}{N-K} \sum_{k=1}^{K} \sum_{i=1}^{n_k} \left\| d_i - c_k \right\|^2}, \tag{21}$$

where n_k denotes the numbers of spots in the kth cluster, c_k the centroid of the kth cluster, and c the global centroid.

- Davies-Bouldin (DB) index: The DB index (Davies and Bouldin [1979]) evaluates clustering quality by measuring the average similarity between each cluster and the cluster most similar to it. It balances intra-cluster compactness and inter-cluster separation. The DB index is always positive, with a lower value indicating better clustering, as it reflects well-separated and compact clusters. Given K clusters, the DB index is calculated as:

$$\text{DB} = \frac{1}{K} \Sigma_{i=1}^{K} \underset{j \neq i}{max} \left(\frac{s_i + s_j}{d_{i,j}} \right), \tag{22}$$

where s_i denotes the average intra-cluster distance for cluster i, $d_{i,j}$ denotes the distance between the centroids of clusters i and j.

Table S5. The range and direction of benchmarks. ↑ indicates higher values represent better performance, ↓ indicates lower values are better.

Metric	Type	Range	Direction
CEMUSA	External	[0, 2]	↓
ARI	External	[-1, 1]	↑
NMI		[0, 1]	↑
Jaccard index		[0, 1]	↑
FMI		[0, 1]	↑
V-measure		[0, 1]	↑
ASW	Internal	[-1, 1]	↑
CHAOS		[0, ∞)	↓
PAS		[0, 1]	↓
CH index		(0, ∞)	↑
DB index		(0, ∞)	↓

Normalized metrics

When evaluating the real spatial clustering results generated by the nine clustering methods (see Supplementary Section 7), the value of each evaluation metric is normalized to $[0, 1]$ to account for differences in their scales and directions, thereby making them comparable. A higher normalized value indicates better clustering performance. The normalization of a metric s is performed as follows:

$$s_{norm} = \begin{cases} \frac{s - \inf_s}{\sup_s - \inf_s}, & \text{if larger metric score indicates better clustering quality,} \\ \frac{\sup_s - s}{\sup_s - \inf_s}, & \text{otherwise.} \end{cases}, \tag{23}$$

where s represents the original score, $\sup_s$ and $\inf_s$ represent its supremum and infimum boundaries, respectively. If $\sup_s$ and $\inf_s$ do not exist, they are replaced by the maximum and minimum values observed among the six methods, respectively.

Supplementary Section 6. Datasets

Our study utilizes four datasets: a BaristaSeq mouse primary visual cortex (mVISp) dataset (slice 1) (Chen et al. [2018]), a 10x Visium human dorsal lateral prefrontal cortex (hDLPFC) dataset (slice 151507) (Maynard et al. [2021]), a 10x Visium human breast cancer (hBC) dataset (slice A1 and H1) (Andersson et al. [2020]), and a MERFISH mouse hypothalamus (mHypo) dataset (tissue section Bregma-0.04) (Moffitt et al. [2018]). The mVISp dataset include 1525 spots and six domain types. The hDLPFC dataset comprises 4,226 spots and seven domain types, while the hBC-A1 dataset and the hBC-H1 dataset include 346 spots and six domain types, and 613 spots and seven domain types, respectively. The mHypo dataset include 5,926 spots and eight domain types. Detailed dataset descriptions and data availability can be found in Table S6.

Table S6. Four ST datasets used in the seven experimental scenarios.

ST dataset	Sample	ST protocol	Resource
mVISp (Chen et al. [2018])	Mouse primary visual cortex	BARISTAseq	`https://spacetx.github.io/`
hDLPFC (Maynard et al. [2021])	Human dorsal lateral prefrontal cortex	10x Visium	`https://research.libd.org/spatialLIBD/`
hBC (Andersson et al. [2020])	Human breast cancer	10x Visium	`https://github.com/almaan/her2st/tree/master`
mHypo (Moffitt et al. [2018])	Mouse hypothalamus	MERFISH	`https://datadryad.org/stash/dataset/doi:10.5061/dryad.8t8s248`

Supplementary Section 7. Spatial clustering methods

Nine methods are utilized to generate real spatial clustering results in our study: **Louvain** (Que et al. [2015]), **Leiden** (Traag et al. [2019]), **CCST** (Li et al. [2022]), **SpaGCN** (Hu et al. [2021]), **STAGATE** (Dong and Zhang [2022]), **GraphST** (Long et al. [2023]), **MNMST** (Wang et al. [2024]), **SEDR** (Xu et al. [2024]), and **Banksy** (Singhal et al. [2024]). The first two methods, Louvain and Leiden, are widely used for clustering cells in scRNA-seq data. **Louvain** builds a spot-spot similarity matrix based on gene expression profiles, and applies multiple clustering algorithms (e.g., k-means, spectral clustering, and Hierarchical clustering), combining their outputs to identify robust and biologically meaningful clusters. **Leiden**, a community detection algorithm, builds a k-nearest neighbor (kNN) graph based on spot similarities in gene expression and refines the initial partitions to produce well-separated subclusters. The remaining four methods are specifically designed for spatial clustering in ST, adopting different mechanisms. **CCST** construct an undirected graph to represent spatial locations and gene expression profiles of spots. It generates both local and global features using graph convolution applied to the original and corrupted graphs. Spot embeddings are then generated through a discrimination task using the Deep Graph Infomax (DGI) approach. **SpaGCN** utilizes a vanilla GCN to generate hybrid spot embeddings that integrates spatial gene expression with associated histology images, enabling the clustering of spots into tissue domains. **STAGATE** models spatial gene expression using a graph and uses a reconstruction-based graph attention network (GAT) to generate low-dimensional spot embeddings, which are then clustered using an off-the-shelf clustering algorithm. **GraphST** models spatial gene expression using a graph augmented with a locally corrupted graph. A Graph Convolutional Network (GCN) is employed to perform self-supervised contrastive learning between the two graphs, yielding spot embeddings for spatial clustering. **MNMST** formulates spatial transcriptomics as a multi-layer network problem, constructing complementary “expression” and “spatial” graphs per section and jointly aligning them into a shared latent space. **SEDR** learns a joint representation by coupling a deep autoencoder for gene expression with a variational graph autoencoder over a coordinate-derived kNN graph. The two embeddings are concatenated and optimized with a combined reconstruction objective, and clustering is refined using a deep embedding clustering (DEC) scheme. **Banksy** introduces neighborhood-augmented features that encode local microenvironments. In addition to intrinsic expression, it computes spatial neighborhood summaries (e.g., local mean expression) and orientation-aware filters (e.g., Gabor/anisotropic features), embedding cells into a product space of “self $\times$ neighborhood” representations followed by scalable graph clustering.

Supplementary Section 8. Detailed descriptions of the sixth and seventh experimental scenario

The sixth experimental scenario evaluates real spatial clustering results from the 10x Visium human breast cancer (hBC) dataset (slice A1) generated by three recent spatial clustering methods (see Supplementary Section 7), including MNMST (Wang et al. [2024]), SEDR (Xu et al. [2024]), and Banksy (Singhal et al. [2024]). As shown in the Figure S6a, MNMST most closely matches the ground truth visually: the invasive cancer region (red) is largely recovered as a single contiguous domain with regular boundaries and minimal spillover. Despite some minor errors, non-cancer compartments are preserved. SEDR is second best: although it captures much of the invasive cancer, the large right region shows a great portion of cancer spots (red) misclassified to connective tissue (blue)—a classic false-negative pattern. This harms both label agreement and the biological severity of mistakes. Banksy performs

worst: a nearly homogeneous region in the ground truth is split into several regular blocks; labels are fragmented and evenly tiled across the tissue, yielding domains that do not correspond to the ground truth structures. Consequently, CEMUSA reflects the visual ranking—MNMST best, SEDR intermediate, Banksy worst —penalizing SEDR's false-negative pattern and strongly penalizing Banksy's spatially implausible over-segmentation. By contrast, internal metrics (e.g., ASW/CH index/DB index) tend to reward Banksy because its partitions are geometrically regular, despite being anatomically incorrect. Conventional externals emphasize overall label matching and often favor SEDR on this slide (e.g., NMI/Jaccard/V-measure), but they do not capture the clinical severity of the right-side false negatives or the difference between SEDR and the more faithful MNMST. Taken together, the experiment illustrates exactly why CEMUSA is informative here: it aligns with the qualitative pathology and differentiates methods by both topology and error severity, not just by aggregate accuracy.

The seventh experimental scenario evaluates real spatial clustering results generated from the MERFISH mouse hypothalamus (mHypo) dataset (tissue section Bregma-0.04) using the same three recent clustering methods (see Supplementary Section 7). The visualizations in Figure S6b reveal that SEDR is visually the most faithful to the ground truth: it largely reflects the relative layout of BST (green) and MPA (blue) and preserves the central V3/PV/PVT compartments (all methods failed to cluster PVH). The symmetric organization and the major cell compartments are correctly clustered, and visually it is the closest to the ground truth. MNMST is second—its main disadvantage is failing to cluster MPA (blue) and mixing MPN (light blue) with BST (green), yielding fuzzier, less separable regions. Banksy performs worst, collapsing most areas into a single BST class and dispersing the remaining types, which contradicts the well-delineated ground truth. Consistently, CEMUSA assigns SEDR the best score, penalizes MNMST's irregular regions and missed MPA, and gives Banksy the worst score. Because this case lacks an error severity aspect, conventional external metrics also favor SEDR and disfavor Banksy, whereas internal indices are inconsistent across methods. Overall, CEMUSA's advantage is that it jointly accounts for label agreement and spatial organization, separating methods that look similar by accuracy alone but differ in anatomical plausibility.

Supplementary Section 9. Detailed results of four real experimental scenarios

Table S7. Evaluation of nine unsupervised spatial clustering methods using real ST data.

Dataset	Method \ Metric	CEMUSA	ARI	NMI	Jaccard index	FMI	V-measure	ASW	CHAOS	PAS	CH index	DB index
hBC-A1 dataset	Louvain	1.200	0.038	0.170	0.132	0.374	0.170	-0.059	0.242	0.775	19.524	9.378
	Leiden	1.168	0.071	0.225	0.175	0.413	0.225	-0.086	0.230	0.757	20.139	6.244
	CCST	1.460	0.066	0.230	0.135	0.405	0.230	0.321	0.145	0.026	315.310	0.858
	SpaGCN	1.230	0.032	0.180	0.143	0.372	0.180	-0.085	0.240	0.777	7.224	12.880
	STAGATE	0.968	0.164	0.221	0.207	0.544	0.221	-0.111	0.191	0.480	25.290	3.226
	GraphST	0.819	0.121	0.219	0.195	0.531	0.219	-0.104	0.202	0.367	28.506	6.885
mHypo dataset	Louvain	1.460	0.092	0.166	0.222	0.299	0.166	-0.141	0.053	0.540	14.147	36.913
	Leiden	1.525	0.101	0.173	0.228	0.308	0.173	-0.140	0.054	0.530	12.065	55.546
	CCST	1.628	0.326	0.508	0.288	0.438	0.508	0.272	0.028	0.005	4072.782	1.055
	SpaGCN	1.439	0.184	0.259	0.301	0.384	0.259	-0.191	0.042	0.336	22.383	17.414
	STAGATE	0.920	0.296	0.489	0.437	0.436	0.489	-0.118	0.030	0.096	265.970	7.067
	GraphST	0.707	0.238	0.421	0.445	0.413	0.421	-0.115	0.032	0.251	220.882	16.523
hBC-A1 dataset	MNMST	0.681	0.198	0.222	0.196	0.612	0.221	0.076	0.155	0.084	92.806	0.907
	SEDR	1.252	0.163	0.319	0.247	0.526	0.319	0.067	0.147	0.052	51.962	4.783
	Banksy	1.479	0.063	0.241	0.155	0.394	0.241	0.325	0.146	0.032	293.174	0.969
mHypo dataset	MNMST	1.493	0.168	0.291	0.302	0.408	0.291	-0.150	0.041	0.254	115.881	14.187
	SEDR	0.819	0.297	0.427	0.450	0.436	0.427	-0.207	0.037	0.221	129.635	15.365
	Banksy	1.667	0.104	0.223	0.281	0.371	0.223	-0.157	0.048	0.309	55.866	27.104

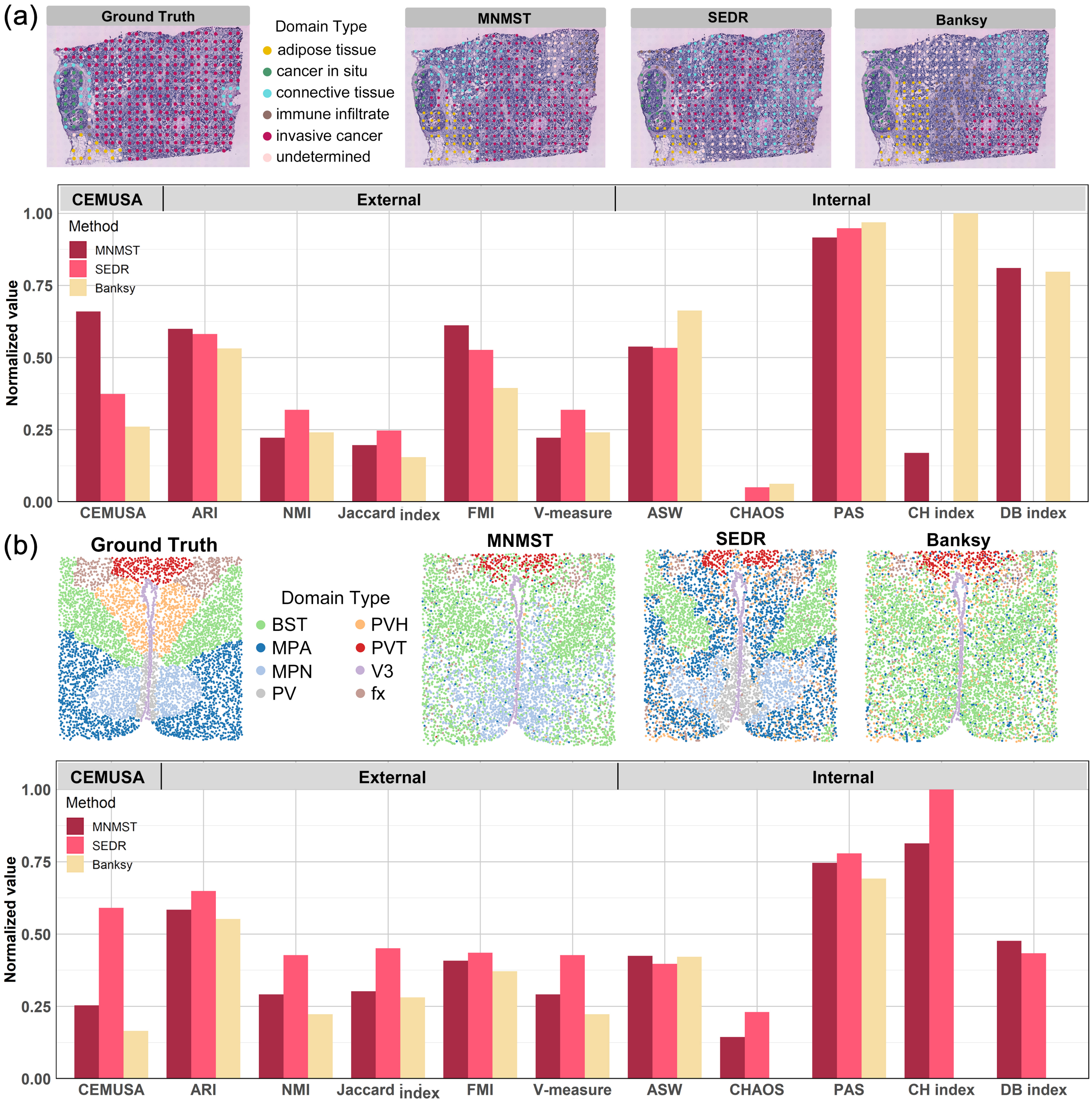

Fig. S6. Evaluation of real spatial clustering results. (a) Evaluating clustering results from the 10x Visium human breast cancer (hBC) dataset (slice A1). The upper panels display the ground truth labeling and real results of recent three spatial clustering methods using the 10x Visium human breast cancer (hBC) dataset (slice A1). The bottom panel presents the normalized scores of CEMUSA and benchmark metrics for these methods. Note that normalized scores always increase with result quality. **(b)** Evaluating clustering results on the MERFISH mouse hypothalamus (mHypo) dataset. The upper panels display the ground truth labeling and real results of recent three spatial clustering methods. The bottom panel presents the normalized scores of CEMUSA and benchmark metrics for these methods. Note that normalized scores always increase with result quality.

References

T. Alexandrov and A. Bartels. Testing for presence of known and unknown molecules in imaging mass spectrometry. *Bioinformatics*, 29(18):2335–2342, 2013.

A. Andersson, L. Larsson, L. Stenbeck, F. Salmén, A. Ehinger, S. Wu, G. Al-Eryani, D. Roden, A. Swarbrick, Å. Borg, et al. Spatial deconvolution of her2-positive breast tumors reveals novel intercellular relationships. *bioRxiv*, pages 2020–07, 2020.

N. Aronszajn. Theory of reproducing kernels. *Transactions of the American mathematical society*, 68(3):337–404, 1950.

F. Batool and C. Hennig. Clustering with the average silhouette width. *Computational Statistics & Data Analysis*, 158:107190, 2021.

C. Berg, J. P. R. Christensen, and P. Ressel. *Harmonic analysis on semigroups*, volume 100. Springer, New York, 1984a.

C. Berg, J. P. R. Christensen, P. Ressel, C. Berg, J. P. R. Christensen, and P. Ressel. General results on positive and negative definite matrices and kernels. *Harmonic Analysis on Semigroups: Theory of Positive Definite and Related Functions*, pages 66–85, 1984b.

Y. Brenier. Polar factorization and monotone rearrangement of vector-valued functions. *Communications on pure and applied mathematics*, 44(4):375–417, 1991.

T. Caliński and J. Harabasz. A dendrite method for cluster analysis. *Communications in Statistics-theory and Methods*, 3(1): 1–27, 1974.

X. Chen, Y.-C. Sun, G. M. Church, J. H. Lee, and A. M. Zador. Efficient in situ barcode sequencing using padlock probe-based baristaseq. *Nucleic acids research*, 46(4):e22–e22, 2018.

T. M. Cover. *Elements of information theory*. John Wiley & Sons, New York, 1999.

D. L. Davies and D. W. Bouldin. A cluster separation measure. *IEEE transactions on pattern analysis and machine intelligence*, (2):224–227, 1979.

K. Dong and S. Zhang. Deciphering spatial domains from spatially resolved transcriptomics with an adaptive graph attention auto-encoder. *Nature communications*, 13(1):1739, 2022.

L. Guo, Z. Hu, C. Zhao, X. Xu, S. Wang, J. Xu, J. Dong, and Z. Cai. Data filtering and its prioritization in pipelines for spatial segmentation of mass spectrometry imaging. *Analytical chemistry*, 93(11):4788–4793, 2021.

M. Halkidi, Y. Batistakis, and M. Vazirgiannis. On clustering validation techniques. *Journal of intelligent information systems*, 17:107–145, 2001.

J. Hu, X. Li, K. Coleman, A. Schroeder, N. Ma, D. J. Irwin, E. B. Lee, R. T. Shinohara, and M. Li. Spagcn: Integrating gene expression, spatial location and histology to identify spatial domains and spatially variable genes by graph convolutional network. *Nature methods*, 18(11):1342–1351, 2021.

I. T. Jolliffe and D. B. Stephenson. *Forecast verification: a practitioner's guide in atmospheric science*. John Wiley & Sons, New York, 2012.

S. Kolouri, Y. Zou, and G. K. Rohde. Sliced wasserstein kernels for probability distributions. In *Proceedings of the IEEE Conference on Computer Vision and Pattern Recognition*, pages 5258–5267, 2016.

J. Li, S. Chen, X. Pan, Y. Yuan, and H.-B. Shen. Cell clustering for spatial transcriptomics data with graph neural networks. *Nature Computational Science*, 2(6):399–408, 2022.

Y. Long, K. S. Ang, M. Li, K. L. K. Chong, R. Sethi, C. Zhong, H. Xu, Z. Ong, K. Sachaphibulkij, A. Chen, et al. Spatially informed clustering, integration, and deconvolution of spatial transcriptomics with graphst. *Nature Communications*, 14(1): 1155, 2023.

K. R. Maynard, L. Collado-Torres, L. M. Weber, C. Uytingco, B. K. Barry, S. R. Williams, J. L. Catallini, M. N. Tran, Z. Besich, M. Tippani, et al. Transcriptome-scale spatial gene expression in the human dorsolateral prefrontal cortex. *Nature neuroscience*, 24(3):425–436, 2021.

J. R. Moffitt, D. Bambah-Mukku, S. W. Eichhorn, E. Vaughn, K. Shekhar, J. D. Perez, N. D. Rubinstein, J. Hao, A. Regev, C. Dulac, et al. Molecular, spatial, and functional single-cell profiling of the hypothalamic preoptic region. *Science*, 362(6416): eaau5324, 2018.

X. Que, F. Checconi, F. Petrini, and J. A. Gunnels. Scalable community detection with the louvain algorithm. In *2015 IEEE international parallel and distributed processing symposium*, pages 28–37. IEEE, 2015.

W. M. Rand. Objective criteria for the evaluation of clustering methods. *Journal of the American Statistical association*, 66 (336):846–850, 1971.

A. Rosenberg and J. Hirschberg. V-measure: A conditional entropy-based external cluster evaluation measure. In *Proceedings of the 2007 joint conference on empirical methods in natural language processing and computational natural language learning (EMNLP-CoNLL)*, pages 410–420, 2007.

L. Shang and X. Zhou. Spatially aware dimension reduction for spatial transcriptomics. *Nature communications*, 13(1):7203, 2022.

V. Singhal, N. Chou, J. Lee, Y. Yue, J. Liu, W. K. Chock, L. Lin, Y.-C. Chang, E. M. L. Teo, J. Aow, et al. Banksy unifies cell typing and tissue domain segmentation for scalable spatial omics data analysis. *Nature genetics*, 56(3):431–441, 2024.

V. A. Traag, L. Waltman, and N. J. Van Eck. From louvain to leiden: guaranteeing well-connected communities. *Scientific reports*, 9(1):1–12, 2019.

Y. Wang, Z. Liu, and X. Ma. Mnmst: topology of cell networks leverages identification of spatial domains from spatial transcriptomics data. *Genome Biology*, 25(1):133, 2024.

H. Xu, H. Fu, Y. Long, K. S. Ang, R. Sethi, K. Chong, M. Li, R. Uddamvathanak, H. K. Lee, J. Ling, et al. Unsupervised spatially embedded deep representation of spatial transcriptomics. *Genome Medicine*, 16(1):12, 2024.
